# Morphological plasticity in a native freshwater fish from semiarid Australia in response to variable water flows

**DOI:** 10.1002/ece3.3167

**Published:** 2017-07-20

**Authors:** Jennifer L. Kelley, Peter M. Davies, Shaun P. Collin, Pauline F. Grierson

**Affiliations:** ^1^ School of Biological Sciences (M092) The University of Western Australia Crawley WA Australia; ^2^ Centre of Excellence in Natural Resource Management The University of Western Australia Albany WA Australia; ^3^ UWA Oceans Institute (M470) The University of Western Australia Crawley WA Australia

**Keywords:** altered water flows, arid zone fauna, group phenotypic composition, morphological divergence, phenotypic plasticity, phenotypic trait change

## Abstract

In fishes, alterations to the natural flow regime are associated with divergence in body shape morphology compared with individuals from unaltered habitats. However, it is unclear whether this morphological divergence is attributable to evolutionary responses to modified flows, or is a result of phenotypic plasticity. Fishes inhabiting arid regions are ideal candidates for studying morphological plasticity as they are frequently exposed to extreme natural hydrological variability. We examined the effect of early exposure to flows on the development of body shape morphology in the western rainbowfish (*Melanotaenia australis*), a freshwater fish that is native to semiarid northwest Australia. Wild fish were collected from a region (the Hamersley Ranges) where fish in some habitats are subject to altered water flows due to mining activity. The offspring of wild‐caught fish were reared in replicated fast‐flow or slow‐flow channels, and geometric morphometric analyses were used to evaluate variation in fish body shape following 3, 6, 9, and 12 months of exposure. Water flows influenced fish morphology after 6 and 9 months of flow exposure, with fish in fast‐flow environments displaying a more robust body shape than those in slow‐flow habitats. No effect of flow exposure was observed at 3 and 12 months. Fishes also showed significant morphological variation within flow treatments, perhaps due to subtle differences in water flow among the replicate channels. Our findings suggest that early exposure to water flows can induce shifts in body shape morphology in arid zone freshwater fishes. Morphological plasticity may act to buffer arid zone populations from the impacts of anthropogenic activities, but further studies are required to link body shape plasticity with behavioral performance in habitats with modified flows.

## 1 INTRODUCTION

The world's waterways are under increasing threat from human activities including impoundments, water abstraction, and the projected effects of climate change on regional hydrological patterns (Dudgeon et al., 2006; Vorosmarty et al., 2010). The impact of altered hydrological regimes on freshwater biota is considered to be particularly severe in arid and semiarid regions because populations tend to be spatially fragmented across the landscape and thus vulnerable to both poor recruitment and local extinction risk (Faulks, Gilligan, & Beheregaray, 2010). For example, climate change models for intermittent streams in the arid American southwest have revealed that the increased frequency of stream drying events will reduce hydrological connectivity and result in a reduction in the probability of dispersal of native fishes (Jaeger, Olden, & Pelland, 2014). Freshwater habitats in arid zones are also under increasing pressure for water resource development, which can alter the natural regime of high flow variability and potentially impact species diversity and ecosystem health (Bunn, Thoms, Hamilton, & Capon, 2006).

The uncertainty associated with the naturally dynamic hydrological regime of the arid zone may have resulted in strong selection for phenotypic plasticity, which could potentially provide some level of resilience to human‐induced flow alterations (Palkovacs, Kinnison, Correa, Dalton, & Hendry, 2011). Phenotypic change can also occur through evolutionary responses to changing or novel selective pressures and can facilitate relatively rapid changes in traits (Ellner, Geber, & Hairston, 2011). However, arid zone species with restricted ranges often show limited population differentiation and low genetic diversity (Faulks et al., 2010; Meffe & Vrijenhoek, 1988). This low genetic diversity may limit the variation upon which selection, and selection for phenotypic plasticity, can act, potentially restricting species' responses to environmental variability. Determining whether human activities induce trait changes, and whether such shifts act to facilitate or prevent ecological change, requires a decoupling of evolutionary responses and trait plasticity (Hendry et al., 2011). However, surprisingly few studies have examined phenotypic plasticity in fishes that inhabit arid or semiarid regions that are already naturally subjected to extreme variation in water flows.

Freshwater fishes exhibit considerable morphological plasticity in response to environmental variables such as water flow (Imre, McLaughlin, & Noakes, 2002; Pakkasmaa & Piironen, 2001), predation risk (Bronmark & Miner, 1992; Eklöv & Jonsson, 2007) and habitat structure (Olsson & Eklöv, 2005; Sass, Gille, Hinke, & Kitchell, 2006). However, it has only recently been revealed that human activities that disrupt natural flow regimes can also contribute to changes in morphological traits (reviewed by Palkovacs et al., 2011). Studies with North American freshwater fishes (e.g., shiners, *Cyprinella* spp., brook silversides, *Labidesthes sicculus,* and bluegill sunfish*, Lepomis macrochirus*) have revealed that water impoundment by dam construction is associated with within‐species morphological divergence; specifically, fish from reservoirs were found to have deeper bodies and smaller heads than those from stream populations (Cureton & Broughton, 2014; Franssen, 2011; Franssen, Harris, Clark, Schaefer, & Stewart, 2012; Haas, Blum, & Heins, 2010). This morphological divergence is consistent with body shape morphologies that are optimized to the flow conditions of the fishes' habitat, suggesting that the differentiation is adaptive; a fusiform body shape is suited to sustained swimming in continuous water flows in streams, while a deep body and wide caudal peduncle are suited to burst swimming performance and maneuvering in a lentic habitat, such as a pond or reservoir (reviewed by Langerhans & Reznick, 2010). The study with red shiners (C. *lutrensis*) revealed that the morphological differences between fish from reservoir and stream populations were maintained in laboratory‐reared fish, suggesting that some of the observed variation in body shape has a genetic basis (Franssen, 2011). Nonetheless, red shiners exhibited morphological plasticity in response to a live predator, suggesting that ecological cues can induce body shape changes in this species (Franssen, 2011). As a number of laboratory experiments have revealed that fishes exhibit morphological plasticity in response to water flows (Fischer‐Rousseau, Chu, & Cloutier, 2010; Imre et al., 2002; Keeley, Parkinson, & Taylor, 2007; Pakkasmaa & Piironen, 2001), it is most probable that other types of human‐induced flow alteration, besides impoundment, result in changes in body shape morphology.

In this study, we conducted a laboratory experiment to investigate the effect of early exposure to water flows on the development of body shape in a native Australian freshwater fish, the western rainbowfish *Melanotaenia australis*. The western rainbowfish is widely distributed across the northwest of Australia, including the semiarid Pilbara region where it occupies a wide variety of freshwater habitats including streams, springs, swamps, and lakes (Allen, Midgley, & Allen, 2002). Western rainbowfish typically grow to a maximum of 10 cm in total length; males are also larger, wider‐bodied, and more brightly colored than females (Allen et al., 2002). The body coloration is characterized by one or two dark midlateral stripes, a red/orange cheek spot, a series of lateral red/orange stripes, and brightly colored fins (Young, Simmons, and Evans, 2011). Rainbowfish are opportunistic feeders, consuming a diet of filamentous algae, aquatic insects and terrestrial vertebrates (Pusey, Kennard, & Arthington, 2004). In their natural habitat, rainbowfish are often found near submerged vegetation (Hattori & Warburton, 2003), but in habitats with flowing water, they are seen feeding on material floating on the surface or in the water column (J. Kelley, personal observation).

**Figure 1:**
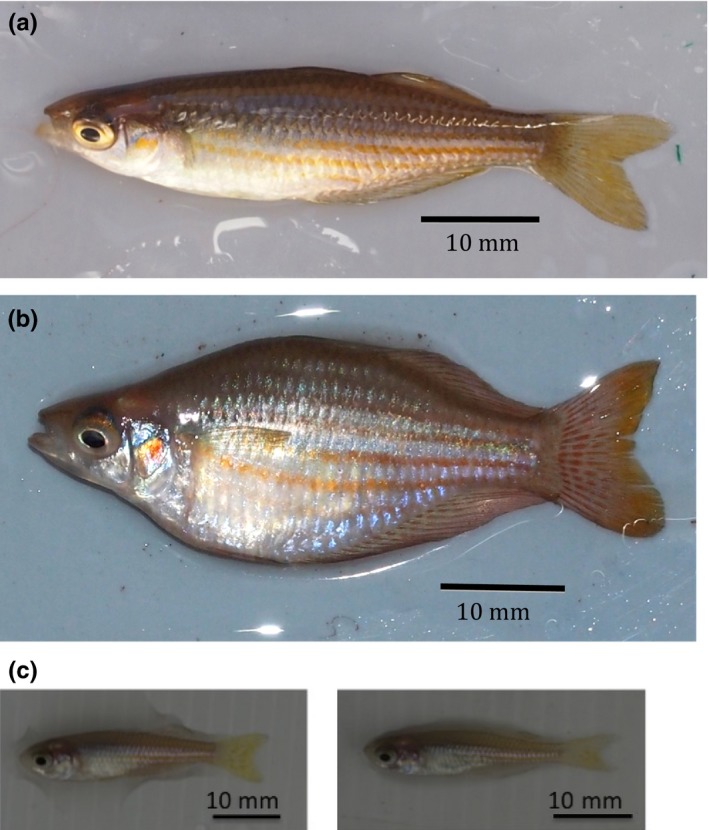
Example of the extreme morphological variation that can be exhibited by the study species, the western rainbowfish (*Melanotaenia australis*). Individuals were collected from Flat Pool in Karijini National Park (a; latitude −22.4776, longitude 118.5567) and Crossing Pool in Millstream National Park (b; latitude −21.5813, longitude 117.5813) in the Pilbara region of northwest Australia. Photographs by Sam Lostrom. Note that these images are for illustrative purposes and the observed morphologies did not arise from the flow treatments used in this study. (c) Morphological variation observed in the current study for fish exposed to 9 months of fast‐flow (left) or slow‐flow (right) rearing treatment

Previous research has revealed that western rainbowfish exhibit considerable variation in body shape (Figure 1), which can be explained by both sexual dimorphism and geographic effects (Lostrom et al., 2015). Interestingly, this previous study found that fish from a site, where flows have been modified for ~9 years due to mining activities (continuous and fast‐flowing water from groundwater discharge), had more streamlined bodies than those from a comparable site nearby (Lostrom et al., 2015). A fusiform body shape is considered to optimize steady swimming in fast water flows, a finding that is consistent with our understanding of swimming biomechanics (Langerhans, 2008; Langerhans & Reznick, 2010). While there is some evidence that morphological differentiation in this species is consistent with the hierarchical genetic structuring of populations (Young, Evans, and Simmons 2011), the extent to which phenotypic plasticity contributes to body shape variation has not previously been considered in this species, or many other arid zone species.

The goals of this study were to (1) investigate whether water flows can contribute to body shape plasticity in a fish from the semiarid zone that is exposed to naturally unpredictable water flows, and (2) to determine whether morphological plasticity might partly explain the divergence in body shape reported previously in fish from a site receiving large volumes of mine discharge water (Lostrom et al., 2015). Here, we based our study on fish collected from a nearby but unmodified catchment with comparable hydrogeomorphology and ecological characteristics. We raised the offspring of wild‐caught fish under fast or slow water flows and used geometric morphometric analyses to evaluate changes in body shape morphology at 3‐month intervals, over a period of 1 year. We anticipated that if body shape is phenotypically plastic in western rainbowfish, fish reared in fast‐flow conditions would exhibit a fusiform body shape (i.e., to optimize sustained swimming performance), while those reared in slow flows would be deeper‐bodied (i.e., suited to burst swimming). If morphological plasticity can be induced by water flow in the laboratory, then a shift in body shape morphology in the wild may act to buffer arid zone fishes from the effects of unnatural and sustained water flows.

## 2 MATERIALS AND METHODS

### 2.1 Study area

Rainbowfish were collected from a natural and hydrologically isolated pool (latitude −23.0098S, longitude 119.6199E) on Coondiner Creek, which forms part of the Fortescue River catchment in the Pilbara region of northwest Australia (Siebers et al., 2015). The Pilbara covers an area of ~500,000 km^2^ and has a semiarid to subtropical climate, with average temperatures ranging from 6–21°C in the winter and 21–39°C in the summer (Lostrom et al., 2015). The average annual rainfall in this region is 300–500 mm, but this is highly variable among years (annual range is between 60 and 800 mm; Bureau of Meterology 2016). Coondiner Creek is typical of many dryland rivers in the region typically consisting of a series of disconnected pools running along a creek bed. The creek flows only in response to intense rainfall events, which are usually associated with cyclonic activity during the Austral summer (December–March). Some pools in this creek are connected to alluvium water and maintained by surface water flow‐through, while others are hydrologically isolated and reliant on rainfall (Fellman et al. 2011; Siebers et al. 2015). Fish sampling was conducted at the end of the dry season in October 2013; thus, little rainfall (<1 mm) had fallen in the proceeding months.

### 2.2 Fish collection and maintenance

Approximately 150 adult western rainbowfish of mixed sex were captured (under Department of Parks and Wildlife license SF009252 and Department of Fisheries Exemption no. 2235) from the pool using a 10‐m seine net (mesh size = 6 mm). Fish were placed in bags containing creek water, zeolite (for removal of ammonia) and Stress Cote^™^ (0.1 ml/L) and immediately packed into polystyrene crates for transport to the freshwater aquarium at The University of Western Australia (Perth). Adult fish were placed in five large (79.5 cm × 49.5 cm × 30.5 cm, depth = 24 cm) mixed sex aquaria where they were maintained on a mixed diet of commercial flake food (AquaOne Tropical Flakes) and *Artemia* nauplii (brine shrimp). Lighting was provided by overhead fluorescent strip bulbs on a 12: 12‐hr light: dark cycle. Aeration was provided using a submersible filter pump. Rainbowfish are “trickle spawners” and exhibit breeding activity all year round, with peak spawning in the wild occurring during the (wet) summer months in the wild (Allen, 1995). Juveniles were obtained by removing all of the adults from a stock tank and allowing the fry to hatch from the gravel substrate, which occurred over the following 7‐ to 10‐day period. Newly hatched fry were fed a mixed diet of *Paramecium* and vinegar eels for the first 4 weeks and *Artemia* nauplii thereafter until 4 months of age. At this age/size, they were deemed suitable for exposure to water flows and were transferred to the flow rearing channels. We collated fry that hatched within a 14‐day period (i.e., combining fry from several tanks) of one another and randomly assigned 40 individuals to each of high‐flow or slow‐flow rearing channels. Fish maintenance and handling was conducted under the Australian code of practice for the care and use of animals for scientific purposes under The University of Western Australia Animal Ethics Committee approval (no. RA/3/100/1176).

### 2.3 Flow channels

**Figure 2:**
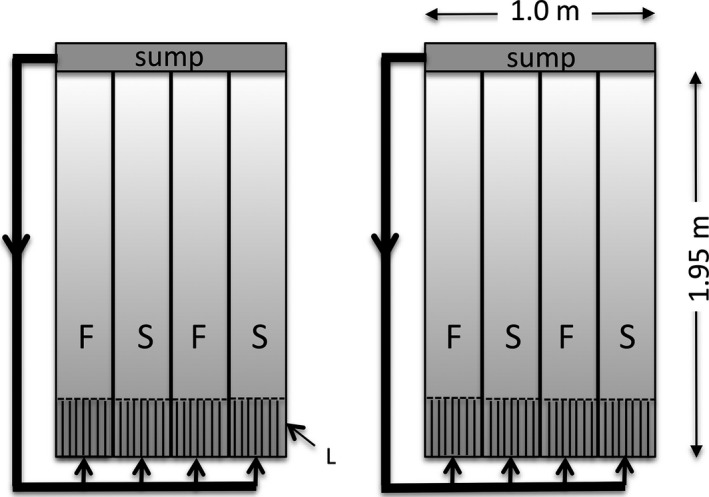
Diagram of apparatus used in this experiment. Aquarium water from each sump was recirculated and directed into one of four flow channels (S: slow flow; F: fast flow). The inflow pipes for each channel were fitted with a tap (indicated by arrows), allowing us to control the water flow speed in each channel. A series of narrow (diameter = 11 mm, length = 22 cm) pipes were placed 10 cm behind the outlet pipe to create laminar flow in each channel (L)

The flow channels comprised two sets of four plastic PVC hemispherical pipes (diameter = 245 mm, length = 1.95 m), each of which formed a closed circulation system, draining into a large (157 cm × 71 cm × 50 cm, filled to a depth of 35 cm) sump containing a 11 kL/h pump (Tunze^™^, Texas, USA) (Figure 2). Polypropylene bioballs (42 mm diameter) were placed in the sump to provide biological filtration. Mesh barriers (2 mm diameter) were placed behind the laminar flow pipes and at the end of each flow channel to contain fish within the channel. We also placed transparent perspex lids (containing a small hole for food delivery; diameter = 33 mm) on each of the channels to prevent fish jumping out. Fish were fed *Artemia nauplii* until 6 months of age and then a mixed diet of commercial flake food (AquaOne tropical flakes) and *Artemia*. This dietary change was applied across all treatments and was necessary to fulfill the nutritional requirements of the developing fish. To allow fish adequate time for feeding and to prevent food being washed away in the water current, the pumps were turned off once a day for 15 min while fish were fed. This short cessation of flow could be problematic if the development of musculature used for foraging is specific to water flow rates. However, leaving the pumps on could potentially confound overall exposure to flow with foraging performance in fast/slow water flows. We did not adjust food quantity according to flow speed, but ensured fish in each channel were fed to satiation. Water temperature in the channels was maintained at 26 ± 1°C, and lighting was provided by two overhead fluorescent strip lights on a 12:12‐hr light:dark cycle. The stocking density at the start of the experiment, when fish were <10 mm in total length, was ~80 fish/m^2^; densities of juveniles in the wild are unknown, but adult densities have been reported from 0.01 to 31.25 fish/m^2^ (Morgan & Gill, 2004). Although we did not closely monitor the fish (to avoid disturbance effects), we did not notice any aggression between individuals or any differences in fish aggression between the flow treatments or during the course of the experiment. Two channels in each circulation system were set for fast flow, and two were adjusted as slow flow. We measured water flow in each of the channels using a Sontek^™^ Flowtracker (an acoustic Doppler velocimeter), which measures flow velocity to an accuracy of 0.0001 ms^−1^. We took five readings in each channel (at variable distances from the outlet pipe) for both X (adjacent to flow direction) and Y (orthogonal to the flow direction) dimensions and calculated the mean and standard error for each. Each water flow reading was measured over a 10‐s period. The average flow speed (mean ± *SE*) for the high‐flow treatment was 0.042 ± 0.0036 ms^−1^ adjacent to the flow (X) and −0.0088 ± 0.0027 ms^−1^ orthogonal to the flow direction (Y). Average speeds for X and Y in the slow channels were 0.0034 ms^−1^ ± 0.0021 and −0.0032 ms^−1^ ± 0.0018, respectively. The flow rate in the fast‐flow channels was at the lower end of the range of water flows recorded for the creek (Weeli Wolli) receiving mine water discharge (mean surface water velocity measured over 30‐s interval: min: 0.03 ± 0.00; max: 0.33 ± 0.02 ms^−1^) (Lostrom et al., 2015).

### 2.4 Fish photography

After 3‐months exposure to water flows (fast or slow flow), a total of 20 fish were randomly selected from each flow channel and photographed using a digital SLR camera (Nikon D7100) fitted with a macrolens (AF/S Nikor 60 mm). Diffuse lighting was used to photograph fish in a small light tent (60 × 60 × 60 cm), lit from the side by two 500W (Arlec, Victoria, Australia) halogen lamps. Fish were photographed out of the water on their right side, using a scale bar and greyscale standard (mini Munsell Colourchecker (MI, USA). As the photography procedure is rapid (<5 s), fish were photographed without using anesthesia. Following each round of photography, five fish were removed, both to reduce the stocking density as fish grew larger and because they were required for another experiment, while the remaining 15 fish were returned to their allocated flow channel. This photography procedure was repeated when fish had been in the flow channels for 6, 9, and 12 months (*n* = 4 photography sessions per flow channel; 80 photographs for each channel). Thus, the number of fish in each channel was successively reduced from *n* = 40 at the start to *n* = 35 (after 3 months), *n* = 30 (after 6 months), *n* = 25 (after 9 months), and *n* = 20 (after 12 months). We counted the number of fish present in each channel every 3 months, to determine whether water flow treatment influenced mortality. Mortality rates were higher in the slow‐flow treatment lanes than in the high‐flow treatment lanes (mean number of deaths/lane; slow flow = 4.38 fish, fast flow = 2.69 fish), but the difference was not significant (2‐sample *t* test: *t*_6_ = 1.29, *p* = .25). The final sample sizes were: 158 for 3 months (fast: *n* = 79, slow: *n* = 79), 160 at 6 months (fast: *n* = 80, slow: *n* = 80), 130 for 9 months (fast: *n* = 67, slow: *n* = 63), and 76 at 12 months (fast: *n* = 35, slow: *n* = 41).

### 2.5 Morphometric analyses of body shape

**Figure 3:**
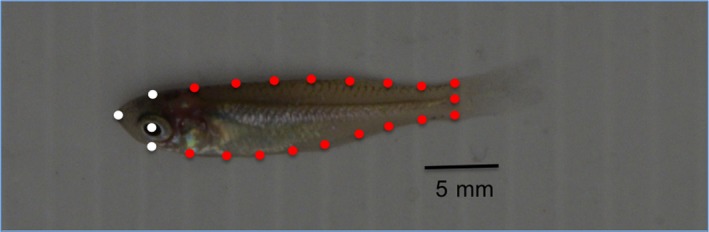
Location of fixed (white) and semisliding (red) landmarks used for geometric morphometric analysis of body shape in juvenile western rainbowfish (*Melanotaenia australis)*. Image shows a fish from the slow‐flow treatment, photographed after 3 months of flow exposure

All TIFF images were first adjusted for spatial scale (in mm), using the scale bar included in each image. Images were then imported into TpsUtil and TpsDig software (available at http://life.bio.sunnysb.edu/morph/morph/) to assign the landmarks to each image for subsequent morphometric analyses (Rohlf, 2005). We placed a total of 22 “landmarks” on each image: 18 semilandmarks and four fixed landmarks. Landmark placement was conducted by a naive experimenter who was not aware of the flow treatment groups. Fixed landmarks were placed in homologous locations on each fish, which were the tip of the upper jaw, the center of the eye, and at the top of the head, directly above and below the eye (Figure 3). Semilandmarks were placed along the curved edges of the body and approximately equidistant between fixed landmarks. Following landmark and semilandmark placements, TpsRelw (Zelditch, Swiderski, & Sheets, 2012) was used to generate the relative warp scores (RWs), which describe morphological variation independent of body size. As our analyses of changes in morphology were not statistically independent (see below), separate sets of RWs were generated for 3, 6, 9, and 12 months. The centroid size, calculated as the square root of the summed distances of each landmark from the centroid position, was used as a measure of body size. The relationship between centroid size and the RWs describes allometry in a body shape; a negative correlation is indicative of negative allometry, while a positive correlation is indicative of positive allometry.

### 2.6 Statistical analyses

We ran four separate independent analyses, one for each developmental stage (i.e., 3, 6, 9, and 12 months). We used independent analyses to avoid pseudoreplication because fish in each treatment were randomly selected for the photography and then returned to their experimental lane; thus, some fish would have been photographed more than once during the course of the experiment. There was some mortality over the 12‐month flow exposure period so the density of fish in each lane became increasingly variable during the course of the experiment. This effect is important to consider as fish growth rates are known to be dependent on stocking density (Refstie, 1977). There is also evidence that conspecific density affects phenotypic plasticity in other taxa, such as amphibians (Davenport & Chalcraft, 2014; Guariento, Carneiro, Esteves, Jorge, & Caliman, 2015). While we ensured flow conditions in each experimental channel were as similar as possible, it is likely that there was some subtle variation in flow dynamics within the four channels comprising each treatment that may further explain any observed morphological variation. To consider the role of these effects, we used MANCOVA, considering the combined relative warps (RW1–RW29) as the dependent variables and lane nested within treatment as a fixed effect. Flow speed (fast or slow) was also entered as a fixed effect, and centroid was included in all models as a covariate. We tested for an interaction between treatment and centroid size and between lane (nested in treatment) and centroid size to test for heterogeneity of slopes and variation in allometry due to treatment or lane effects. Statistical analyses were conducted using the open source computer software program R, version 3.2.2 (R Development Core Team, 2016).

We used canonical variate analysis (CVA) in the software program MorphoJ version 1.06d (Klingenberg, 2011) to visualize changes in mean shape associated with the channels and the treatments. We first performed a procrustes analysis to align the landmarks by the principal axes and remove any variation associated with size. We examined pairwise differences between the channels using permutation tests (10,000 permutations) of the Mahalanobis distance to compare within‐treatment and among‐treatment effects on mean morphology.

## 3 RESULTS

### 3.1 Variation in body shape morphology

A total of 40 relative warps (RWs) were obtained, of which the first 29 (RW1‐RW29) had eigenvalues >1 and explained 100% of the total variation in the data. Inspection of the thin plate spines revealed the main sources of morphological variation. The first relative warp (RW1) explained 20%–30% of the total variation in the data and was associated with narrowing of the body, particularly in the ventral (i.e., abdomen) region (Table 1). RW2 and RW3 explained 20%–26% and 11%–14% of the total variance in body shape morphology, respectively, and were associated with body deepening in the dorsal area, thickening of the caudal peduncle (RW2), and anterior body deepening (RW3) (Table 1). RW4 and RW5 explained only 5%–9% of body shape variation and described a forward tilt of the anterior body (RW4) and head widening (RW5) (Table 1). These are generalized patterns as the warps described slightly different aspects of morphology at each developmental stage; for example, RW1 was associated with anterior body deepening during the early stages of flow exposure (3–6 months), but described caudal peduncle thickening and shortening after 9–12 months of flow treatment (Table 1). The remaining warps (RW6–RW29) are not described and explain a small proportion of the total variance in shape.

**Table 1:** Percentage variation and cumulative percentage of variation in body shape explained by relative warps 1–5 for each developmental stage

Developmental stage	*n*	RW	%	Cumm %	+ RW scores	‐ RW scores
3 months	158	RW1	30.16	30.16	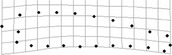	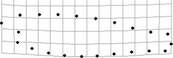
		RW2	24.5	54.65		
		RW3	13.89	68.54		
		RW4	8.72	77.26		
		RW5	5.70	82.95		
6 months	160	RW1	35.2	35.2	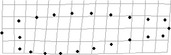	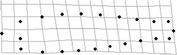
		RW2	21.6	56.8		
		RW3	10.34	67.14		
		RW4	8.12	75.26		
		RW5	5.26	80.52		
9 months	130	RW1	40.4	40.4	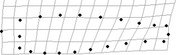	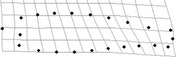
		RW2	19.89	60.3		
		RW3	11.43	71.73		
		RW4	8.25	79.98		
		RW5	5.71	85.69		
12 months	76	RW1	33.84	33.84	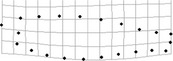	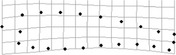
		RW2	25.76	59.60		
		RW3	13.24	72.83		
		RW4	8.43	81.26		
		RW5	5.16	86.43		

The number of individuals used to generate the relative warps (*n*) is also shown, along with the morphological variation associated with positive and negative scores for RW1

### 3.2 Effect of flow on overall body shape development

The MANCOVA models revealed a significant effect of lane nested within treatment and a significant effect of centroid (a measure of body size) on overall fish morphology (described by the RWs) at each developmental stage (Table 2). There was a significant interaction between lane (nested within treatment) and centroid following 6 months of flow exposure, suggesting that there was variation in allometry according to lane, but this interaction was not significant at the other developmental stages (Table 2). The MANCOVAs showed an overall effect of treatment (water flow speed) on fish morphology after 6 and 9 months of flow exposure, but not after 3 or 12 months of exposure to different water flow speeds (Table 2). There was no significant interaction between centroid and treatment for any developmental stage (3 months: *F*_29, 122_ = 0.86, *p* = .68; 6 months: *F*_29, 124_ = 1.23, *p* = .22; 9 months: *F*_29, 94_ = 0.58, *p* = .95; 12 months: *F*_29, 40_ = 1.36, *p* = .18). There was also no effect of water flow speed on centroid size at any stage of flow experience (*t* tests: 3 months: *t*_155.8_ = −0.54, *p* = .59; 6 months: *t*_158_ = −0.43, *p* = .67; 9 months: *t*_118.5_ = −0.45, *p* = .66; 12 months: *t*_74_ = 0.42, *p* = .68), suggesting that growth rates were not affected by flow treatment.

**Table 2:** MANCOVA results displaying the overall effect of treatment (fast or slow water flow), lane (nested in treatment), centroid, and the interaction between lane (nested in treatment) and centroid on overall morphology (combined RW scores)

Developmental stage (months)	Effect	Df	Wilks	*F*	*p*
3	Treat	29, 123	0.748	1.43	.094
	Treat (lane)	58, 246	0.340	3.04	**<.001**
	Centroid	29, 123	0.309	9.49	**<.001**
	Treat (lane) × Centroid	29, 123	0.621	1.14	.244
6	Treat	29, 125	0.568	3.273	**<.001**
	Treat (lane)	58, 250	0.506	1.751	**.002**
	Centroid	29, 125	0.313	9.459	**<.001**
	Treat (lane) × Centroid	58, 250	0.511	1.719	**.002**
9	Treat	29, 95	0.635	1.886	**.012**
	Treat (lane)	58, 190	0.210	3.871	**<.001**
	Centroid	29, 95	0.397	4.978	**<.001**
	Treat (lane) × Centroid	58, 190	0.601	0.949	.583
12	Treat	29, 41	0.499	1.422	.148
	Treat (lane)	58, 82	0.169	2.021	**.002**
	Centroid	29, 41	0.265	3.930	**<.001**
	Treat (lane) × Centroid	58, 82	0.275	1.282	.150

A significant interaction between centroid and lane (nested in treatment) indicates that allometric growth differs among lanes within the same treatment. Significant effects are shown in bold. The interaction between lane and treatment did not have a significant effect in any of the models and was therefore removed.

**Figure 4:**
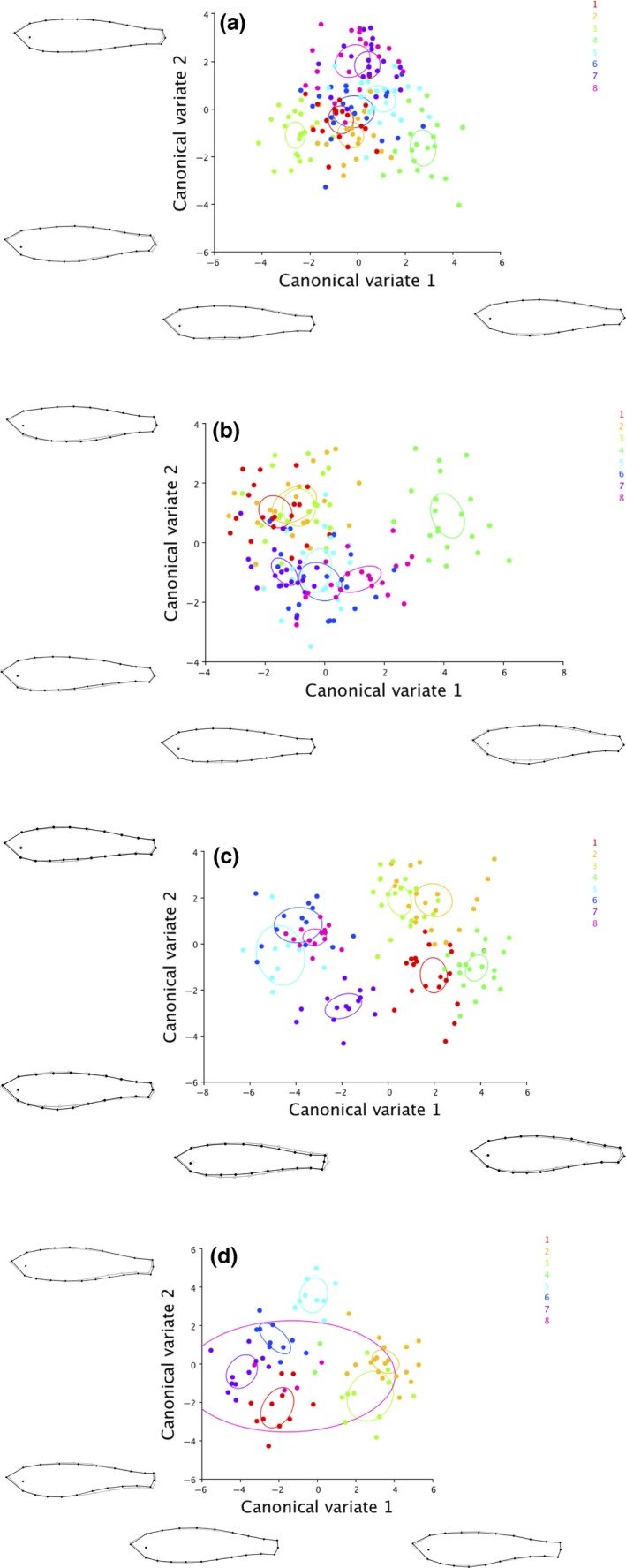
Plot of canonical variates (CV1 and CV2) for fish following 3 months (a), 6 months (b), 9 months (c), and 12 months (d) of exposure to fast or slow water flow speeds. Ellipses represent 95% confidence intervals of the mean for each treatment channel. Legend indicates lane numbers (even: fast flow; odd: slow flow). Changes in shape described by the CVs are illustrated for positive and negative scores (Mahalanobis distance scaled according to maximum and minimum on each axis). Note the large 95% CI for lane 8 at 12‐month development and that the 95% CI is missing for lane 4 due to small sample size (*n* = 2 fish)

The canonical variate analysis revealed that there was considerable morphological separation according to the rearing lane at all four developmental stages (Figure 4a–d). Morphological separation by lane was most apparent for CV1, which was associated with deepening of the body and caudal peduncle for positive CV1 scores and body narrowing for negative CV1 scores (Figure 4a–d). Variation in the second canonical variate (CV2) was also linked with body depth and with body curvature, describing an upward/downward facing head (Figure 4a–d). Mahalanobis distances ranged from 2.5 to 8.89 during fish development, and the range of Mahalanobis distances was greater between flow treatments than within treatments (i.e., among channels) and increased over the course of the fish's development in both treatment groups (Table 3). The permutation tests for the Mahalanobis distances revealed significant differences in mean shape between all pairwise combinations of lanes, both within and between treatments (all *p* < .017) with the exception of two fast‐flow lanes at 12 months (lane 4 and lane 8; *p* = .065) (Table 3). The Mahalanobis distances corroborate the findings of the CVA; at 6 months of age, fish in lane 4 (fast‐flow treatment) showed high levels of morphological differentiation relative to other channels in the same treatment, while fish in lane 8 (fast‐flow treatment) at 12 months showed similar levels of morphological separation within and between treatments (Table 3, Figure 4b, d).

**Table 3:** Mahalanobis distances, describing the shape change per unit of within‐group variation, measured among channels and between flow treatments

(a)							
	2	4	6	8	1	3	5
4	4.14						
6	3.16	4.1					
8	3.88	4.85	3.49				
1	**2.99**	**4.15**	**2.81**	**3.19**			
3	**3.70**	**5.43**	**3.67**	**4.28**	3.08		
5	**3.07**	**3.38**	**2.78**	**2.85**	2.72	4.29	
7	**3.81**	**4.45**	**3.05**	**2.43**	3.50	4.45	2.56

(b)							
	2	4	6	8	1	3	5
4	5.6						
6	3.94	5.16					
8	4.04	4.41	3.72				
1	**3.44**	**6.03**	**3.6**	**4.60**			
3	**2.60**	**5.34**	**3.82**	**3.80**	3.05		
5	**3.24**	**5.05**	**2.96**	**3.12**	3.69	3.04	
7	**3.32**	**5.92**	**3.21**	**3.59**	3.32	3.00	2.5

(c)							
	2	4	6	8	1	3	5
4	2.24						
6	6.92	8.31					
8	6.22	7.57	4.41				
1	**4.15**	**2.86**	**6.82**	**6.08**			
3	**3.12**	**5.15**	**5.69**	**5.20**	4.13		
5	**7.54**	**8.89**	**4.29**	**3.88**	7.18	6.53	
7	**6.38**	**6.94**	**5.75**	**5.19**	5.35	5.66	5.29

(d)							
	2	4	6	8	1	3	5
4	7.07						
6	6.46	6.00					
8	7.27	7.62*	6.30				
1	**6.67**	**6.96**	**4.56**	**6.20**			
3	**5.37**	**6.79**	**7.28**	**7.25**	6.24		
5	**6.46**	**7.07**	**5.23**	**6.98**	6.65	6.39	
7	**7.79**	**7.26**	**4.51**	**5.73**	4.34	7.92	6.41

Variation in morphology was assessed following exposure to fast or slow water flows for 3, 6, 9, and 12 months. Shaded boxes represent high‐flow channels, and Mahalanobis distances in bold are between‐treatment comparisons in fish morphology (fast versus slow flow). All Mahalanobis distances were significantly different between channels (*p* < .05) with the exception of lane 4 and lane 8 to 12 months (marked with an asterisk).

## 4 DISCUSSION

Our findings suggest that body shape morphology is a labile trait in the western rainbowfish that can be altered through early exposure to low/moderate water flows (0.042 ± 0.004 ms^−1^) over a relatively short time frame. Juvenile rainbowfish that were exposed to flowing water for 6–9 months developed a more robust form (i.e., anterior body widening) than those exposed to slow flows, but the effects of flow exposure were not apparent at early (3 months) or at later stages of development (12 months). We also found significant morphological variation within flow treatments, at all stages of flow experience. These results suggest that morphological traits in an arid zone species can exhibit phenotypic plasticity, which may allow them to rapidly respond to the extreme and highly unpredictable hydrology of this region.

The fish used in our study were the first generation of laboratory‐born fish (to exclude the effect of previous experience) and originated from an intermittent stream in a semiarid region where interannual variation in rainfall exceeds 100%. Although our sample stream typically exists as a series of isolated pools with no connectivity (i.e., flow) between pools, our findings suggest that within a few months of continuous flow, fish can adjust their body shape morphology. In our study region, mine water discharge for some 9 years has modified the hydrology of a nearby stream (of comparable ecology) from intermittent to continuous flow (Dogramaci, Firmani, Hedley, Skrzypek, & Grierson, 2015). Populations of western rainbowfish within this stream have thrived and expanded (A. Storey, personal communication), providing evidence that arid zone fishes can adjust to extreme variation in the hydrological environment, including that caused by human activities. While there is some evidence that western rainbowfish in the creek receiving discharge have more fusiform bodies than is typical for the subcatchment (Lostrom et al., 2015), flow conditions in the laboratory (which were relatively uniform) are very unlikely to replicate conditions in the wild (e.g., affected by substrate, vegetation); thus, different morphological responses are not unexpected. Furthermore, our study focused on the morphological responses of a single population to high and low water flows; thus, our results cannot necessarily be generalized to other populations or arid zone fishes without sufficient replication. Nonetheless, our study presents an important first step in investigating morphological plasticity in semiarid freshwater fishes and predicting the potential impacts of modified flow regimes in these regions.

Our finding that fish reared in fast‐flowing waters developed more robust bodies than those reared in slow‐flowing waters contrasts with the general expectation that fast water flows should induce a fusiform body shape to increase streamlining and reduce hydrodynamic drag. However, a number of other studies have also found that fish reared in fast‐flowing waters tend to develop more robust body shapes (reviewed by Langerhans, 2008; Langerhans & Reznick, 2010). Indeed, discrepancies in findings among different studies suggest that the morphological response to water flow tends to be species specific; for example, Peres‐Neto and Magnan (2004) found that head dimensions varied with flow speed in Arctic charr, but there was no effect of flow on these morphological characteristics in brook charr. Similarly, Pakkasmaa and Piironen (2001) showed that Atlantic salmon (*Salmo salar)* had deeper bodies in high water flows and streamlined bodies in slow flows, while brown trout (*Salmo trutta*) exhibited the opposite response. An increased body depth may not necessarily cause increased drag (Schaefer, Lutterschmidt, & Hill, 1999); thus, the relationship between morphology, water flow, and swimming performance may not always be predictable.

We observed a significant (within‐treatment) channel effect throughout the experiment. While we measured flow at the start of the experiment and were careful to maintain water levels such that flow rates were constant over the duration of the experiment, small‐scale flow variation among the channels is likely. If fish are sensitive to flow variation on a small scale, then they may exhibit a plastic response that is specific to the particular flow environment. This is an interesting possibility that warrants further investigation. We did not periodically measure flow rates within each channel during the course of the experiment (this would have entailed considerable disturbance to the fish), and therefore, we were not able to investigate the relationship between within‐channel flow variation and morphological change. It is possible that fish morphology differed within channels of the same treatment before the experiment commenced (i.e., time 0); however, given our efforts to randomly allocate juveniles to rearing channels, this is unlikely. The lack of an effect of flow on morphology after 12 months of flow exposure might be explained by the periodic (every 3 months) reduction in fish density. However, given that fish were approaching the onset of sexual maturity at 12 months (some were beginning to gain coloration), and the level of sexual dimorphism in body shape in this species (Lostrom et al., 2015), it is very likely that the later stages of the experiment (i.e., 12 months) were influenced by sex differences in developmental morphology (which are yet to be described in this species), rather than by exposure to water flows.

A large number of other environmental factors, besides water flow, can induce changes in morphology in fishes, such as diet (Schaefer et al., 1999), temperature (Andersson, 2003; Day & McPhail, 1996) and exposure to olfactory predator cues (Sfakianakis, Leris, Laggis, & Kentouri, 2011). For example, Arctic charr *(Salvelinus alpinus)* that are fed with zooplankton (live *Daphnia*) developed deep bodies, while those fed (frozen) chironomids developed slimmer bodies and larger heads with more downward pointing mouths (Frommen et al., 2011). While all fish were exclusively fed *Artemia* for the first six months of this experiment and then fed on a mixed diet of commercially prepared flake food and *Artemia* for the remaining 6 months (to ensure adequate nutrition and health during the course of development), diet may have differentially affected fish in the fast‐flow treatment, perhaps because a wider body increases maneuverability and facilitates foraging on live prey items. We attempted to minimize these potential differences and standardize food intake by shutting off water flow during the 15‐min daily feeding period. Most studies of morphological plasticity consider a single factor (e.g., water flow, predator cues) in isolation; thus, the potential for traits to interact and influence morphological variation warrants further attention.

In summary, our findings suggest that there is considerable flow‐induced morphological plasticity in the western rainbowfish. As body shape morphology is often tightly coupled with fitness‐related traits, it is likely that a labile morphology has allowed this species to colonize a variety of freshwater habitats and persist in the extreme hydrological conditions prevalent in the arid zone. Arid regions are under increasing pressure from human activities such as agriculture and mining, and to some extent, a rapid and plastic response to hydrological variability may act to buffer species against these impacts. Nonetheless, there are key traits (e.g., the ability for widespread dispersal) that allow fishes to survive in the arid zone, and the disruption of key hydrological events that maintain genetic connectivity may threaten population persistence. Understanding the limits of a species' ability to cope with hydrological variation remains paramount for managing the effects of altered flow regimes on wild populations.

## DATA ARCHIVING STATEMENT

Supporting data for this paper is available online at http://dx.doi.org/10.4225/23/593f38eb2624f.

## CONFLICT OF INTEREST

None declared.
